# Bile acids differentially regulate longitudinal smooth muscle contractility in everted mouse ileum

**DOI:** 10.1096/fba.2024-00044

**Published:** 2024-06-17

**Authors:** Peace N. Dike, Krishnakant G. Soni, Diana S. Chang, Geoffrey A. Preidis

**Affiliations:** ^1^ Division of Gastroenterology, Hepatology and Nutrition, Department of Pediatrics Baylor College of Medicine and Texas Children's Hospital Houston Texas USA

**Keywords:** Gpbar1 protein, mouse, receptors, cytoplasmic and nuclear, receptors, muscarinic, smooth muscle

## Abstract

Bile acids regulate gastrointestinal motility by mechanisms that are poorly understood. Standard isolated tissue bath assays might not recapitulate in vivo physiology if contractile responses to certain bile acids require direct application to the intestinal mucosa. We sought to determine the feasibility of quantifying longitudinal smooth muscle contractile responses to bile acids from intact segments of everted mouse ileum. Ileum from adult female C57BL/6J mice was isolated, gently everted over a notched metal rod, and mounted in tissue baths. Individual bile acids and agonists of bile acid receptors were added to the baths, and longitudinal smooth muscle contractile responses were quantified by isometric force transduction. Ursodeoxycholic acid robustly increased contractile responses in a dose‐dependent manner. Deoxycholic acid stimulated contractility at low doses but inhibited contractility at high doses. Chenodeoxycholic acid, glycocholic acid, and lithocholic acid did not alter contractility. The dose‐dependent increase in contractility resulting from the application of ursodeoxycholic acid was recapitulated by INT‐777, an agonist of the Takeda G protein‐coupled receptor 5 (TGR5), and by cevimeline, a muscarinic acetylcholine receptor agonist. Agonists to the nuclear receptors farnesoid X receptor, glucocorticoid receptor, pregnane X receptor, vitamin D receptor, and to the plasma membrane epidermal growth factor receptor did not modify baseline contractile patterns. These results demonstrate that gentle eversion of intact mouse ileum facilitates the quantification of longitudinal smooth muscle contractile responses to individual bile acids. Prokinetic effects of ursodeoxycholic acid and low‐dose deoxycholic acid are replicated by agonists to TGR5 and muscarinic acetylcholine receptors.

## Graphical abstract

Bile acids regulate gastrointestinal motility by poorly understood mechanisms. In standard isolated tissue bath assays, bile acids cannot contact the intestinal mucosa. Our adapted technique quantifies contractile responses to bile acids in segments of everted ileum. Contractility increases dose‐dependently in response to ursodeoxycholic acid and agonists of Takeda G protein‐coupled receptor 5 (TGR5) and muscarinic acetylcholine receptors. Deoxycholic acid stimulates contractility at low doses but inhibits contractility at high doses. This method may help define mechanisms underlying differential effects of bile acids on ileal contractility.

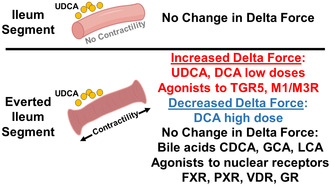

## 1 INTRODUCTION

Bile acids are end products of cholesterol catabolism that facilitate lipid digestion and absorption. Bile acids also influence gastrointestinal motility and fluid and electrolyte transport by selectively activating receptors in the plasma membrane and nucleus and modifying ion channels.^1^ In the colon, bile acids generally are prokinetic. Oral or rectal administration of the primary bile acid chenodeoxycholic acid (CDCA) increases the frequency of propagating pressure wave sequences and accelerates transit.^2^, ^3^, ^4^ Conversely, bile acids can slow transit through the small bowel,^5^, ^6^ contributing to the “ileal brake” that helps optimize digestion. Understanding the mechanisms by which bile acids regulate intestinal motility is important, given that altered bile acid homeostasis is implicated in colonic motility disorders.^7^ For example, fecal bile acid concentrations are elevated in many patients with diarrhea‐predominant irritable bowel syndrome (IBS) or functional bowel disorder with diarrhea^8^, ^9^ and are deficient in subsets of patients with constipation‐predominant IBS.^10^

Some of the prokinetic effects of bile acids in the colon are attributed to their interactions with the plasma membrane Takeda G protein‐coupled receptor 5 (TGR5),^11^ which is expressed in mucosa, muscularis externa, and enteric ganglia throughout the gastrointestinal tract.^12^ Different bile acids have varying affinities to TGR5,^13^, ^14^ and this is one potential explanation of why different bile acids can affect gut motility to various degrees.^1^ Additional receptors and pathways that mediate these responses remain under investigation.^15^ Furthermore, little is known of the mechanisms by which bile acids regulate motility in the small bowel. These knowledge gaps persist in part because stimulatory contractile responses to bile acids have been difficult to model ex vivo. Spontaneous phasic contractions of isolated longitudinal smooth muscle and of full thickness colon are inhibited by 100 μM deoxycholic acid (DCA) in a TGR5‐dependent manner, whereas 100 μM ursodeoxycholic acid (UDCA) does not affect contractility in these model systems.^11^, ^12^ One potential explanation for the lack of observed prokinetic effects of UDCA in these assays is that direct application of bile acids to the intestinal mucosa could be required to positively stimulate longitudinal smooth muscle contractility. The purpose of this Method and Protocol was to determine the feasibility of quantifying longitudinal smooth muscle contractile responses to bile acids from intact segments of everted mouse intestine in which the serosal surface is fully exposed to the tissue bath.

## 2 MATERIALS AND METHODS

### 2.1 Preparations of everted mouse ileum

The Baylor College of Medicine IACUC approved all aspects of this study. C57BL/6J mice, originally sourced from Jackson Laboratory, were obtained from the Center for Comparative Medicine at Baylor College of Medicine. Female mice 8–12 weeks old were used given that many disorders of gut–brain interaction are more prevalent in women than men and to minimize potentially confounding effects of very young or old age on intestinal motility. After euthanization in isoflurane, the distal third of small bowel was harvested and luminal contents were removed by gently flushing with 10% glycerol in Krebs solution (in mmol/L: 120.9 NaCl, 5.9 KCl, 2.5 CaCl_2_, 14.4 NaHCO_3_, 1.2 NaH_2_PO_4_, 1.2 MgCl_2_, 11.5 glucose). Ileum was everted by adapting a previously published protocol for quantifying jejunal glucose transport.^16^ A 2‐mm diameter stainless steel rod with a groove just proximal to the rounded tip was inserted into the proximal end of the ileum. Moistened surgical thread was tied in a knot to secure the proximal ileum to the groove. Tissue was gently everted over the knot until the entire luminal surface was exposed to buffer. The knotted portion of ileum was then cut away and the rod was removed. Tissue remained submerged in Krebs solution throughout dissection. Four to six mice were used per experiment, and each experiment was repeated at least twice with separate groups of mice to ensure rigor.

### 2.2 Ex vivo contractility measurements

The distal most 1 cm of ileum was tied at both ends and suspended in an organ bath for force transduction measurements per our protocol.^17^ Baths were filled with 25 mL Krebs solution and infused continuously with 95% O_2_/5% CO_2_ gas. Bovine serum albumin (BSA, 0.04% w/v) was added to minimize swelling of everted tissue. Isometric force was monitored by an external force‐displacement transducer connected to a PowerLab recorder (ADInstruments). After 30 min of equilibration to 0.4 g tension, baseline contractility was recorded for 5 min. A 10 mmol/L stock solution of each drug (Table 1) was prepared and diluted accordingly in PBS. Motor responses to increasing doses of drug, first 0.1 μmol/L followed by 1, 10, and 100 μmol/L, were recorded for 5 min after each dose. Contractile magnitude was calculated using LabChart software (ADInstruments) as the difference between the mean force of a test dose and the mean force recorded at baseline. Force transduction data were analyzed by two‐way repeated measures ANOVA with Tukey's multiple comparison test to determine differences between treatments. *T*‐tests were used to compare the means of two normally distributed data sets. All statistics were performed using Prism 10 (GraphPad Software).

**TABLE 1:** Reagents used in ex vivo organ bath experiments.

	Drug	Vendor	Catalog #	Stock solution prepared in
Positive control	Carbachol	MilliporeSigma, Burlington, MA, USA	1092009	PBS
Bile acids	Chenodeoxycholic acid	MilliporeSigma	220411	Ethanol
	Deoxycholic acid	MilliporeSigma	30970	Ethanol
	Glycocholic acid	MilliporeSigma	360512	Ethanol
	Lithocholic acid	Cayman Chemicals, Ann Arbor, MI, USA	20253	Ethanol
	Ursodeoxycholic acid	Cayman Chemicals	15121	Ethanol
Receptor agonists	Calcitriol (vitamin D receptor)	Cayman Chemicals	71820	DMSO
	Cevimeline (Muscarinic M1/M3 receptor)	Cayman Chemicals	17802	PBS
	Epidermal growth factor (epidermal growth factor receptor)	Miltenyi Biotec, Bergisch Gladbach, Germany	130‐097	PBS + 1% BSA
	Fexaramine (farnesoid X receptor)	Cayman Chemicals	17369	DMF
	INT‐777 (Takeda G protein‐coupled receptor TGR5)	MedChemExpress, Monmouth Junction, NJ, USA	HY15677	Ethanol
	Methylprednisolone (glucocorticoid receptor)	Cayman Chemicals	15013	DMF
	Rifampicin (Pregnane X receptor)	Cayman Chemicals	14423	DMF

*Note*: The EGF used in this study has ED_50_ ≤ 1.25 ng/mL, corresponding to an activity of ≥0.8 × 10^6^ IU/mg.

Abbreviations: BSA, bovine serum albumin; DMF, dimethylformamide; DMSO, dimethylsulfoxide; PBS, phosphate‐buffered saline.

## 3 RESULTS

### 3.1 Everted distal ileum facilitates the study of contractile responses by bile acids

**FIGURE 1:**
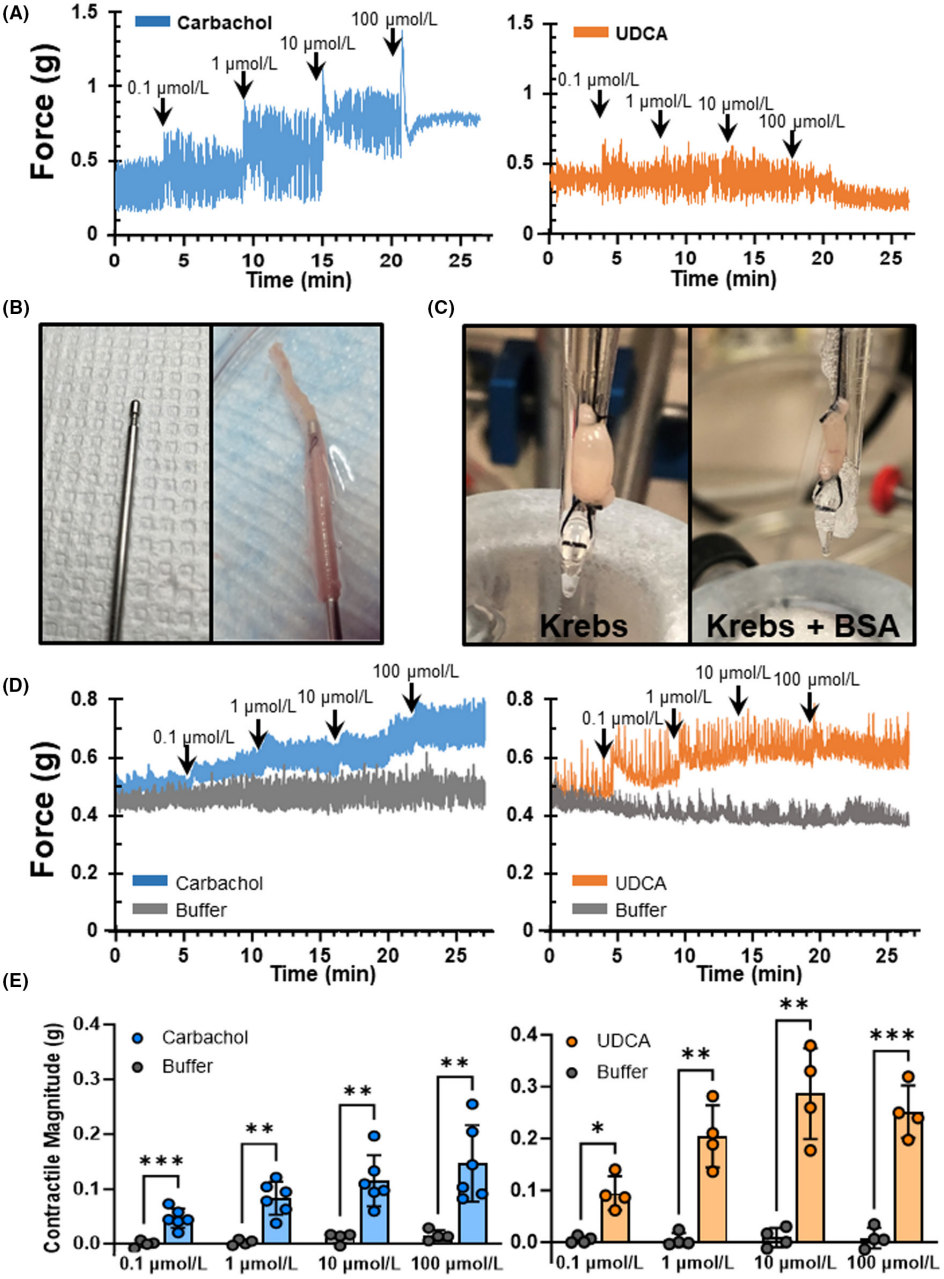
Adaption of an isolated tissue bath assay facilitates direct application of contractile stimuli to the ileal mucosa. (A) Representative force transduction tracings of non‐everted ileum illustrating equilibration at baseline and after exposure to increasing concentrations of drug reveal responsiveness to carbachol but not UDCA. (B) Ileum was everted by suturing the proximal end to a grooved metal rod and gently pulling the tissue over the knot to expose the mucosa. (C) Fully everted ileum tied at both ends and suspended in an organ bath swells upon submersion in Krebs buffer; the addition of 0.04% w/v bovine serum albumin preserves tissue viability. (D,E) Representative force transduction tracings and delta force calculations reveal dose‐dependent longitudinal smooth muscle contractile responses of everted ileum to both carbachol and UDCA, demonstrating the feasibility of this adapted isolated tissue bath technique. UDCA, ursodeoxycholic acid; bars represent mean + SD; *N* = 4–6 mice per group; ***p < 0.001; **p < 0.01; *p < 0.05.

Non‐everted segments of distal ileum responded robustly to carbachol but did not respond to UDCA (Figure 1A). We suspected this lack of effect was due to the bile acid being unable to access the mucosal surface. Thus, we gently everted ileum over a custom grooved metal rod (Figure 1B) to expose the mucosa to the organ bath. Everted ileum swelled in Krebs solution, but the addition of 0.04% w/v BSA prevented swelling and preserved tissue viability (Figure 1C). After adding BSA, everted ileum responded to both carbachol and UDCA in a dose‐dependent fashion (Figure 1D,E).

### 3.2 Ursodeoxycholic acid stimulates and deoxycholic acid inhibits contractility

**FIGURE 2:**
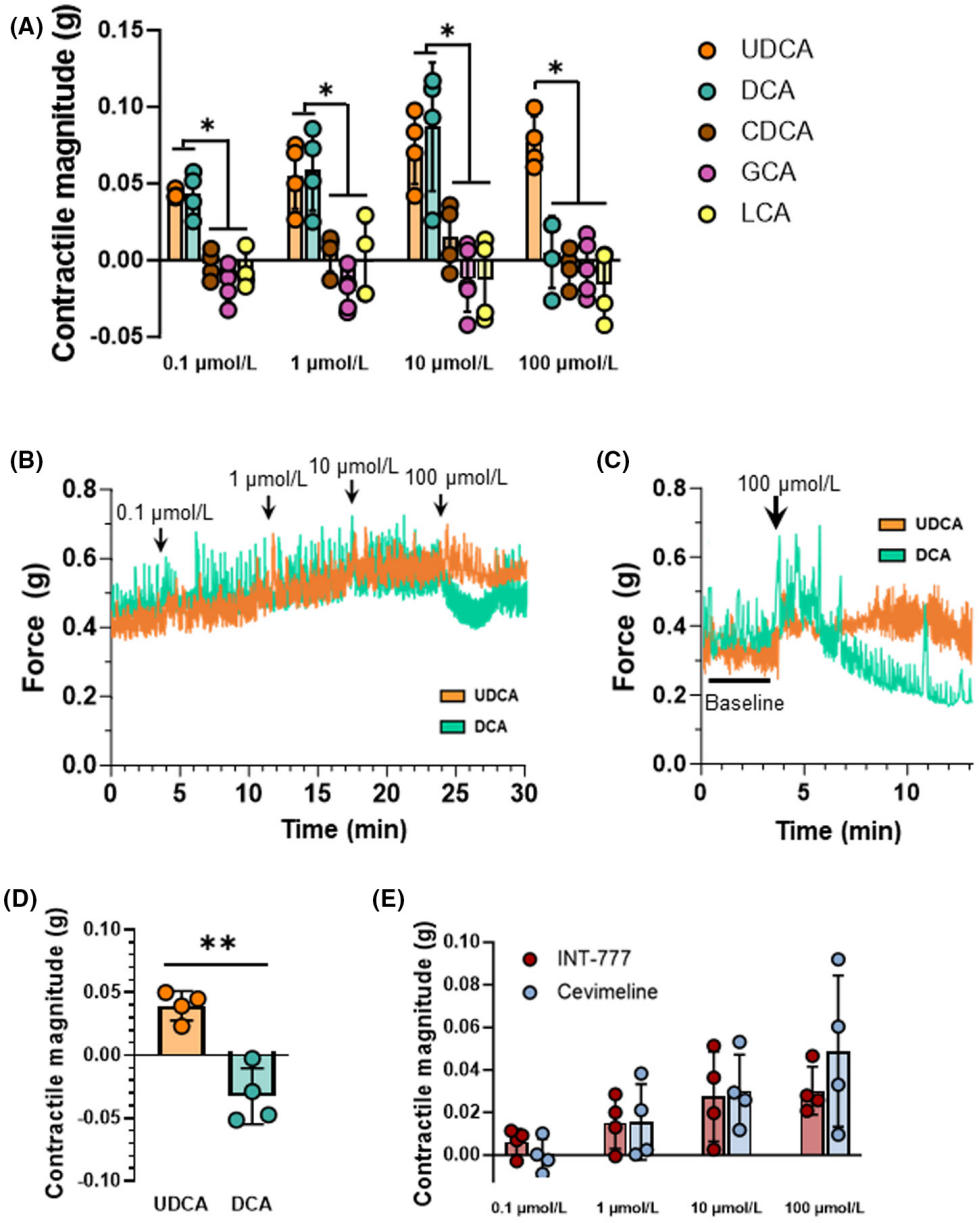
Everted mouse ileum reveals differential effects of bile acids on longitudinal smooth muscle contractility. (A) UDCA stimulates contractile responses at doses spanning the physiologic range, whereas DCA increases contractility at doses of 0.1, 1.0, and 10 μmol/L but inhibits contractility at 100 μmol/L. CDCA, GCA, and LCA do not alter contractile forces in this system. (B) Representative force transduction tracings showing equilibration at baseline and after 0.1, 1.0, 10, and 100 μmol/L of UDCA or DCA. (C,D) Confirmatory experiments illustrate differential responses to a single dose of 100 μmol/L UDCA or DCA. (E) Dose‐dependent increases in contractile responses similar to UDCA are observed following application of the TGR5 agonist INT‐777 or the muscarinic acetycholine receptor agonist cevimeline. Agonists to other receptors known to interact with bile acids (farnesoid X receptor, glucocorticoid receptor, pregnane X receptor, vitamin D receptor, and epidermal growth factor receptor) do not alter contractility (data not shown). CDCA, chenodeoxycholic acid; DCA, deoxycholic acid; GCA, glycocholic acid, LCA, lithocholic acid; UDCA, ursodeoxycholic acid; bars represent mean + SD; ** *p* < 0.01; * *p* < 0.05; *N* = 4–5 mice per group.

Next, we sought to determine whether five of the most abundant bile acids differentially affect ileal contractility at physiologic concentrations (Figure 2A). We confirmed that UDCA increased contractile responses dose‐dependently. Force transduction tracings for DCA were similar to UDCA at doses of 0.1, 1, and 10 μmol/L. However, 100 μmol/L DCA inhibited contractions (Figure 2B). Neither CDCA, glycocholic acid, nor lithocholic acid affected contractility. In confirmatory experiments, we tested a single high dose of UDCA or DCA (Figure 2C). In accord with our initial results, 100 μmol/L of UDCA stimulated and 100 μmol/L of DCA inhibited ileal contractility (Figure 2D; *p* = 0.011).

### 3.3 Agonists of TGR5 and muscarinic acetylcholine receptors induce contractility

Finally, we sought to determine whether we could mimic the opposing effects of UDCA and high‐dose DCA on everted ileum by applying agonists to receptors known to interact with bile acids in the intestine.^15^ Agonists of the nuclear receptors farnesoid X receptor, pregnane X receptor, vitamin D receptor, and glucocorticoid receptor, and of the plasma membrane epidermal growth factor receptor, did not alter contractility (data not shown) similar to CDCA, GCA, and LCA. However, INT‐777, an agonist to TGR5, and cevimeline, a parasympathomimetic agonist to muscarinic acetylcholine receptors, recapitulated the stimulatory effects of UDCA and low‐dose DCA (Figure 2E). None of these receptor agonists at physiologic doses inhibited contractility similar to high‐dose DCA. All together, these data support the feasibility of studying differential contractile responses to bile acids and bile acid receptor agonists using intact, everted segments of mouse intestine.

## 4 DISCUSSION

Mechanistic studies into how bile acids stimulate intestinal contractions are needed to better understand pathophysiology in subsets of gut motility disorders.^7^ In this Method and Protocol, we demonstrate the feasibility of a modified tissue bath assay to measure contractions induced by stimuli applied to intestinal mucosal surfaces. Whereas some drugs including cholinomimetics induce contractions irrespective of tissue configuration, other stimuli including UDCA require exposure to the mucosa, a compartment that in non‐everted tissue is excluded from the organ bath by sutures.

This technique generated the first evidence to our knowledge of a positive stimulatory effect of UDCA on ileal contractility, and of a biphasic response to DCA consisting of increased contractility at low doses and inhibition at 100 μmol/L. Rats gavaged with 5, 10, or 26 μmol/L DCA exhibit dose‐dependent delays in gastric emptying and small bowel transit,^18^ and DCA inhibits neural contractions elicited by electrical field stimulation of colonic smooth muscle strips.^19^ Recent studies report that 1, 10, and 100 μmol/L DCA inhibits the frequency of spontaneous phasic contractions and decreases tension in the non‐everted mouse colon in organ baths and that these effects require TGR5; UDCA on the other hand does not alter contractility.^11^, ^12^ Although UDCA accelerates oroileal transit in adults with gallstone disease,^20^ we are unaware of studies that model the positive stimulatory effects of UDCA in mice. Our finding challenges the general notion that bile acids inhibit motility in the small bowel, and suggests that contractile responses to some bile acids might depend on whether exposure occurs at the mucosal surface or the serosal surface.

Although we recapitulated the prokinetic effects of UDCA and low‐dose DCA on mouse ileum with agonists to TGR5 and muscarinic acetylcholine receptors, none of the receptor agonists we tested reproduced the inhibitory effect of high‐dose DCA. Computer models predict DCA to function as an antagonist to formyl‐peptide receptors, a class of G protein‐coupled receptors^21^; this possibility requires further testing. It was unsurprising that agonists to nuclear bile acid receptors did not alter contractions, given that classic nuclear receptor responses involve transcriptional changes that take place over a much longer duration of time than our ex vivo experimental system facilitates. Although our studies rule out rapid non‐transcriptional effects of nuclear bile acid receptors as causing immediate contractile responses, we cannot exclude the possibility of prolonged effects of bile acids resulting from nuclear receptor activation in vivo.

This experimental technique has limitations, including the additional technical skill required to evert but not otherwise disrupt the intestine. It is likely that even gentle manipulation of the tissue introduces artifacts, and the possibility of variable tissue damage during eversion could contribute to the observed high variance. Alternative ex vivo approaches include infusing bile acids into the lumen for spatiotemporal mapping^22^ and applying bile acids to the apical surface of flat sheet colon preparations for force‐displacement measurements.^11^ Additionally, no region of the mouse gastrointestinal tract was as amenable to tissue eversion as distal ileum; thus, further modifications may be needed to apply this technique to colon and proximal small bowel so that effects of bile acids on smooth muscle contractility throughout the gastrointestinal tract can be more completely understood.

In conclusion, everted intact intestinal segments can be used to study contractile responses to bile acids and other stimuli when direct application to the mucosal surface is desired. Using this adapted organ bath approach will add to our growing knowledge of bile acid‐specific effects on small bowel contractility.

## AUTHOR CONTRIBUTIONS

P. Dike, K. Soni, D. Chang, and G. Preidis conceived and designed the research; P. Dike, K. Soni, and D. Chang performed the research and acquired the data, P. Dike, K. Soni, D. Chang, and G. Preidis analyzed and interpreted the data. All authors were involved in drafting and revising the manuscript.

## FUNDING INFORMATION

National Institute of Diabetes and Digestive and Kidney Diseases, USA, Grant/Award Number: K08 DK113114 to GAP, R03 DK129495 to GAP, R01 DK133301 to GAP, and T32 DK007664 training grant to PND; and the Public Health Service, USA, Grant/Award Number: P30 DK056338, which funds the Texas Medical Center Digestive Diseases Center.

## DISCLOUSRES

The authors declare no conflicts of interest.

## Data Availability

No genomic datasets were generated or analyzed during the current study. The data that support the findings of this study are available in the Section 2 and 3 of this article.
